# Mechanical Properties of Polyurethane Adhesive Bonds in a Mineral Wool-Based External Thermal Insulation Composite System for Timber Frame Buildings

**DOI:** 10.3390/ma14102527

**Published:** 2021-05-13

**Authors:** Ewa Sudoł, Ewelina Kozikowska

**Affiliations:** Construction Materials Engineering Department, Instytut Techniki Budowlanej, 00-611 Warszawa, Poland; e.kozikowska@itb.pl

**Keywords:** external thermal insulation systems, mechanical properties of bonds, polyurethane adhesive, timber frame building, bond strength, shear properties

## Abstract

This paper aims to provide a preliminary assessment of polyurethane adhesive applicability as an alternative to conventional cement-based adhesives used to fix thermal insulation materials to substrates concerning mineral wool-based external thermal insulation composite systems. Currently, polyurethane adhesives are only used in expanded polystyrene-based ETICS. This study discusses the suitability of polyurethane adhesive for ETICS with lamella mineral-wool for timber frame buildings. Bond strength, shear strength and shear modulus tests were conducted. In addition, microstructure and apparent density were analysed. Mechanical properties were analysed in terms of the influence of substrate type and thermal and moisture conditions, taking into account solutions typical for sheathing on timber frame (oriented strand boards (OSB), fibre-reinforced gypsum boards (FGB) and cement-bonded particleboards (CPB)), as well as limit conditions for adhesive application. It was found that PU adhesive can achieve adhesion, both to MW and OSB, and FGB and CPB at ≥80 kPa, which is considered satisfactory for PU adhesives for EPS-based ETICS. Favourable shear properties were also obtained. There was no significant effect of sheathing type on the properties considered, but the influence of temperature and relative humidity, in which the bonds were made, was spotted. The results obtained can be considered promising in further assessing the usefulness of PU adhesives for MW-based ETICS.

## 1. Introduction

External thermal insulation composite systems (ETICS) are among the most popular methods for improving the energy efficiency of buildings in Europe [1]. The first ETICS were installed in the 1960s in Germany and later in Switzerland and Austria [2]. The breakthrough for ETICS came in the early 1970s when, as a consequence of the oil crisis, energy prices rose, resulting in considerations concerning the need to minimise heat loss in buildings [3]. The growth dynamics of the insulation sector in Europe in the 1990s was boosted by the accession to the European Union of new countries, which, while meeting their obligations to comply with EU regulations, soon implemented energy efficiency policies. Currently, Central and Eastern Europe, with countries such as Poland, Germany, Austria, the Czech Republic, Slovakia, Lithuania, Latvia and Estonia, are the undisputed leaders in the ETICS sector. In this region, about 120–130 million m^2^ of building walls are insulated annually. This is more than half of the forecasted European volume of about 215–230 million m^2^. Turkey holds the dominant position concerning individual countries, and Poland, with 40 million m^2^ per year, is in the second place [1,2].

ETICS includes the thermal insulation material fixed to the substrate and the top finishing layer made directly on site, without an air gap or intermediate layers. For many years, expanded polystyrene (EPS) and mineral wool (MW) have been the most commonly used insulation materials in ETICS, followed by other factory-made thermal insulation products such as extruded polystyrene (XPS), polyurethane foam (PUR), phenolic foam (PF) and expanded cork (ICB) [1,4]. The ETICS finishing layer consists of a reinforcement layer made of a base coat with glass fibre mesh embedded and a finishing coat (renders). Some systems also contain key coating, primers and decorative coats [1,5,6].

**Figure 1:**
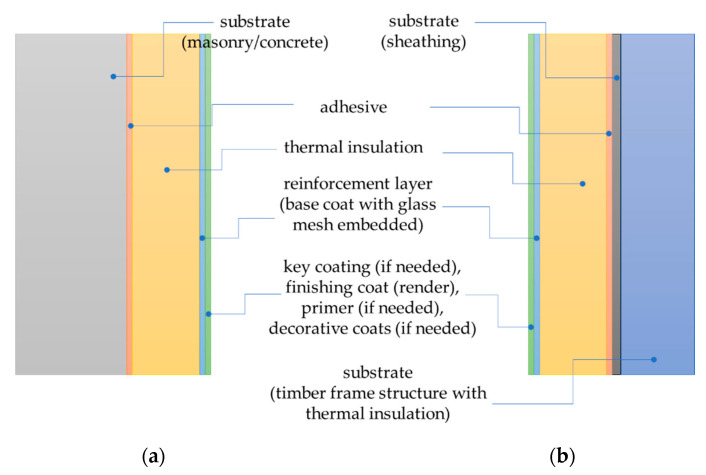
ETICS on (**a**) masonry or concrete wall, (**b**) on wood frame wall. Example of purely bonded ETICS.

ETICS is used as external thermal insulation to the walls of buildings. It can be used on new or existing (retrofit) vertical walls [5]. They can also be used on horizontal or inclined surfaces that are not exposed to precipitation. In Europe, most commonly on masonry walls constructed from units of clay, concrete, calcium silicate, autoclaved aerated concrete laid using mortar and concrete walls made of concrete are either cast on site or as prefabricated panels (Figure 1a) [2]. More and more frequently, ETICS is also being used to insulate walls in timber frame buildings [7]. Then, the thermal insulation material is fixed to the external wall sheathing (Figure 1b), which is usually made of oriented strand boards (OSB), fibre-reinforced gypsum boards (FGB) or cement-bonded particleboards (CPB), less often of fibre-reinforced cement boards or gypsum plasterboards [6,7,8].

Based on the method of fixing the thermal insulation material to the substrate, purely bonded ETICS, bonded ETICS with supplementary mechanical fixings, mechanically fixed ETICS with supplementary adhesive and purely mechanically fixed ETICS are distinguished [5,6]. Fixing the thermal insulation material to the substrate is typically carried out using cement-based adhesive. This applies both to ETICS for use on masonry or concrete walls and timber frame buildings. The exception is some EPS-based ETICS [5,6,9,10]. The construction industry is a dynamic field that constantly has new needs. Therefore, academics and manufacturers’ continually seek to develop new materials and technologies that can be used as effective alternatives to conventional solutions [11]. As far as ETICS is concerned, attempts are being made to use polyurethane adhesives as an alternative to conventional cement-based adhesives for fixing thermal insulation materials other than EPS to substrates. This is in line with the trend observed for many years of wide, on site use of polyurethane foam in construction [12,13]. Apart from standard applications such as roof insulation [14] or window and door fitting [15], on-site foaming polyurethane adhesives are used to bond masonry elements in wall construction [16,17]. Polyurethane adhesives have numerous advantages, such as effective wetting of most substrates; interaction with substrates through polar interactions (hydrogen bonding); and relatively low molecular weight/small molecular size, which allows them to permeate porous substrates and to form covalent bonds with substrates that have active hydrogen atoms [18]. They can be applied at low temperatures [12,13,19] and require no preparation activities on site. They are applied directly from a pressurised container to the material to be fixed. Foaming of polyurethane occurs spontaneously in contact with water present in the materials to be fixed. The foaming agent is carbon dioxide formed by the reaction of water with isocyanate groups [12,20].

As mentioned, polyurethane adhesives for fixing insulation material in ETICS are limited to EPS systems. This is due both to formal considerations and to the lack of a sufficient research basis. Placing ETICS on the market, like several other construction products, is regulated by the construction products regulation (CPR) [21], which establishes harmonised conditions for the marketing of construction products. The assessment of the suitability of use of ETICS is carried out through European or national technical assessment. It is subject to testing and assessing properties that affect the object’s compliance with the basic requirements. Methods and criteria for assessing the essential characteristics of ETICS are fixed on masonry, and concrete walls are defined in EAD 040083-00-0404 [5] amended ETAG 004 [22] in October 2020. Concerning ETICS for use in timber frame buildings, EAD 040089-00-0404 applies [6]. Both of these documents consider using polyurethane adhesive in ETICS only for bonding EPS to the substrate. For such an application, the issue of testing methodology, taking into account the specifics of an on-site foaming polyurethane adhesive, has been systematised [5,23]. This makes it impossible to obtain, by means of a standard procedure, a European Technical Assessment (ETA) for an ETICS where polyurethane adhesive is used for fixing thermal insulation materials other than EPS and thus makes it challenging to place this system on the EU market.

Apart from expanded polystyrene, the second most common thermal insulation material used in ETICS is mineral wool. In Central Europe, the share of ETICS with EPS is about 84% and with MW about 12%, while in the rest of Europe, it ranges from 60 to 88% and from 9 to 25%, respectively [1,2]. Standard ETICS applications are rock wool in the form of factory-made boards as defined in EN 13162 [24], in which the fibres are either dispersed (standard boards) or oriented parallel to each other and perpendicular to the slab surface (lamella boards). Properties of MW boards used in ETICS are presented in Table 1.

For bonded systems and bonded systems with supplementary mechanical fixing, the adhesion of the individual ETICS layers, including the adhesive bonding to both the substrate and the insulation material, as well the shear properties of bonds are crucial in terms of fulfilment of the fourth basic requirement ‘safety in use’ [5,6,25,26]. Although several papers have been devoted to the properties of ETICS, looking at a wide spectrum of properties of individual components and their influence on the essential characteristics of the system [1,3,25,27,28,29,30], including adhesive bonding [31,32,33,34], the authors’ attention has been directed towards cement-based adhesive systems. It has been found that bond strength between cement-based adhesive and the concrete, after 28 days under laboratory conditions, can achieve values from 250 kPa to 1000 kPa [31,33], while after 28 days under laboratory conditions and 2 days in water bond strength decreases to 80 kPa [5]. The bond strength between cement-based adhesive and the thermal insulation material strongly depends on the type of insulation material and the model of damage [31,32]. A review of the literature has shown that bond strength between cement-based adhesive and EPS ranges from 80 kPa to 270 kPa [31,33,34] and bond strength between cement-based adhesive and MW achieved values ranges from 30 kPa to 80 kPa [31,32]. Cohesive damage in the insulation material was usually observed [31,32,33,34]. The bond strength of polyurethane adhesives in EPS-based ETICS achieved at least 80 kPa [5,6,9,10]. In general, the physical and mechanical properties of polyurethane foam, including polyurethane adhesives, are closely related not only to the rigidity of the polymer matrix but also to the size of the cells and their structure [10,35,36,37,38]. Structures with larger cells are characterised by lower apparent density and lower mechanical properties [37,38]. An increase of the water content intensifies the foaming process, leading to an increased cell size [20,39,40]. In wide bonds, carbon dioxide has the ability to form larger bubbles, resulting in a more porous structure [13,35].

This study aims to assess the applicability of polyurethane adhesive as an alternative to conventional cement-based adhesives for lamella mineral wool boards in ETICS for timber frame building. Bond strength, shear strength and shear modulus tests were conducted. In addition, SEM analysis of the adhesive structure in the bond, as well as apparent density, was performed. These properties were analysed in terms of the influence of substrate type and thermal and moisture conditions, taking into account solutions typical for sheathing on timber frame: oriented strand boards (OSB), fibre-reinforced gypsum boards (FGB) and cement-bonded particleboards (CPB), as well as limit conditions for adhesive application.

## 2. Materials and Methods

### 2.1. Adhesive

A single-component polyurethane adhesive, intended for ETICS, a standard for fixing EPS to masonry and concrete substrates, was used for the study. The adhesive was manufactured in industrial conditions as a commercial product. Polyether polyol was used. Organic amine catalyst served as a catalyser to promote blowing reaction. Silicone surfactants were used to stabilise the foam. Polymeric diphenylmethane diisocyanate (PMDI) was used to cure the foam. The components were placed in a standard pressurised container. As a chemical blowing agent, carbon dioxide was generated via the reaction of water included in the substrate, thermal insulation material and the environment with isocyanate groups. The adhesive was applied using a dedicated gun. When sprayed, the adhesive was in the form of a low-pressure foam. The foam adhesive working properties, determined according to [23], are shown in Table 2.

### 2.2. Mineral Wool

**Figure 2:**
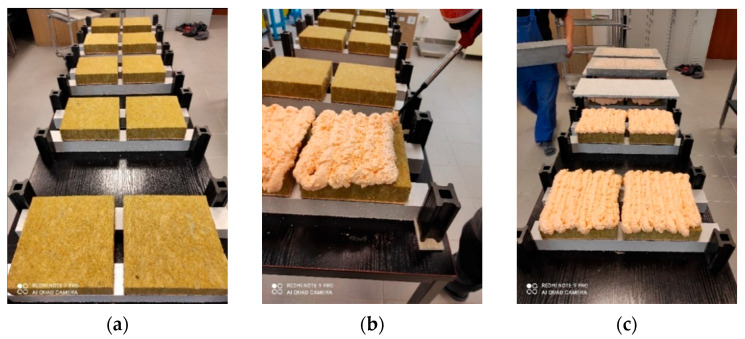
Preparation of samples for bond strength tests (**a**) MW prepared for adhesion, (**b**) application of adhesive to MW, and (**c**) bonding.

Mineral wool lamella boards code MW-EN 13162-T5-DS(70,-)-DS(70,90)-CS(10\Y)40-TR80-WS-WL(P)-MU1, i.e., with perpendicular tensile strength ≥80 kPa, compression strength ≥40 kPa, and other properties described in Table 1, was used for sample preparation. The apparent density of MW was 73 ÷ 88 kg/m^3^, with an average of 82 kg/m^3^. The dimensions of the boards were 1000 mm × 500 mm × 50 mm. They had no coatings or facing in the form of fabric, veil, foil, etc., (Figure 2a), and 200 mm × 200 mm × 50 mm samples were cut from them.

### 2.3. Substrates

The substrates used were boards designed for load-bearing elements that can be used in wet conditions, including wall sheathing for timber frame buildings. Substrates used:

- 18 mm thick oriented strand boards (OSB) with properties appropriate for OSB-3, according to EN 13986 [41];
- 12 mm thick fibre-reinforced gypsum boards (FGB) with properties appropriate for DEFH1IR, according to ETA-14/0312 [42];
- 16 mm thick cement-bonded particleboards (CPB) with properties appropriate for type 634-2, according to EN 13986 [41].

300 mm × 600 mm samples, with their thicknesses maintained, were cut from them.

What is more, 50 mm thick concrete slabs, made at a water to cement ratio of 0.45:0.48, perpendicular tensile strength ≥1.5 MPa and moisture content ≤3% of the total weight, complying with EAD 040083-00-0404 [5], were used as a reference substrate.

### 2.4. Scanning Electron Microscopy (SEM)

The morphology of the polyurethane bonds’ cross-section surface was examined with a field-emission scanning electron microscope (SEM) Sigma 500 VP (Carl Zeiss Microscopy GmbH, Köln, Germany) that renders high-resolution images at low accelerating voltage. The samples were gold-coated before scanning to provide an electrically conductive surface. The accelerating voltage was 10 kV to avoid degradation of the sample. The observations were carried out at 100× magnification.

The study was carried out on a material with 8 mm thick bonds of OSB/23/50/8, OSB/25/30/8, OSB/5/-/8, OSB/25/90/8 and 15 mm OSB/23/50/15 (Table 3). The microstructures of polyurethane bonds were observed on samples cut out perpendicularly to the bonds surface. The samples were cut out with a scalpel at room temperature.

### 2.5. Apparent Density

The test was carried out on cured polyurethane adhesive samples obtained from adhesive bonds made with the OSB described in point 2.3. Five series of 100 mm × 100 mm samples were prepared—in laboratory conditions (23 ± 2 °C, 50 ± 5%), at high temperature and low relative humidity (25 ± 2 °C, 30 ± 5%), at high temperature and high relative humidity (25 ± 2 °C, 90 ± 5%), and at low temperature (5 ± 2 °C) and the resulting RH. The OSB were stored for 24 h in the conditions determined for the adhesive application. The adhesive itself was stored for 24 h in laboratory conditions, which resulted from the manufacturer’s recommendations on the storage conditions. The adhesive was applied to one of the boards in a serpentine pattern. At 180 ± 10 s, the boards were joined together to form sets with distance pieces and screw clamps in which the adhesive bond was 8 ± 1 mm thick or 15 ± 1 mm thick (only in laboratory conditions). The sets were cured for 48 h; 50 mm × 50 mm × 6 mm samples were cut out with a knife afterwards.

The apparent density of the cured polyurethane adhesive was tested according to ISO 854:2006 [43]. The samples were weighed on analytical scales and measured with Vernier calliper. The apparent density of the polyurethane adhesive was calculated as an average mass/volume ratio.

### 2.6. Bond Strength

For bond strength tests, substrate in accordance with point 2.3 was used. Five series of samples were prepared, in different thermal and moisture conditions—in laboratory conditions (23 ± 2 °C, 50 ± 5%), at high temperature and low relative humidity (25 ± 2 °C, 30 ± 5%), at high temperature and high relative humidity (25 ± 2 °C, 90 ± 5%), and at low temperature (5 ± 2 °C) and the resulting RH (Table 3). The abovementioned adhesive application conditions were adopted based on the product manufacturer’s guidelines on the permissible boundary conditions for its use, which fit the concept of ETICS-dedicated polyurethane adhesives tests [5,23].

**Figure 3:**
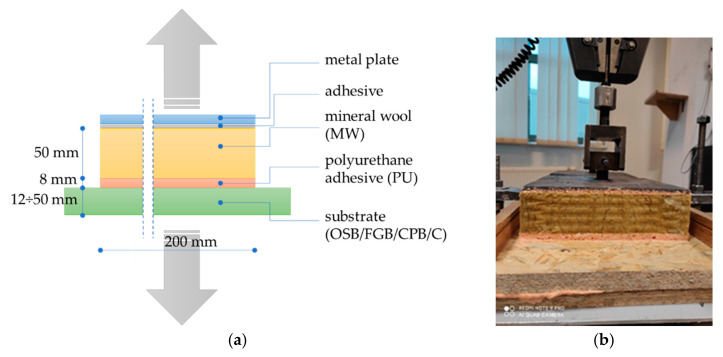
Bond strength test: (**a**) sample scheme, (**b**) sample from OSB/23/50/8 series under load in the testing machine.

Prior to sample preparation, OSB, FGB, CPB, and concrete and MW boards were stored for 24 h under conditions programmed for adhesive application. The adhesive itself was stored for 24 h in laboratory conditions. The adhesive was applied to the mineral wool in the serpentine pattern (Figure 2b). The mineral wool was bonded to the substrate with an open time of 180 ± 10 s., creating 8 ± 1 mm thick bonds with the use of spacers. In laboratory conditions, series with 15 ± 1 mm thick bonds were also prepared. Samples were cured for 24 h under adhesive application conditions. To ensure that the bond thickness remained constant during curing, spacers were maintained, and a load of 15 kg was applied (Figure 2c). At the end of curing, the excess adhesive in the form of squeeze out was cut off, forming 200 mm × 200 mm × 8 mm or 200 mm × 200 mm × 15 mm adhesive bonds (Figure 3a).

Bond strength test involved determining the maximum tensile stress of an adhesive bond with a force acting perpendicular to its face. The method is often used in the testing of construction adhesives [44]. It was derived from TR46 [23]. Tests were conducted under laboratory conditions immediately after the samples were cured under specific conditions (Table 3). The test was carried out using a computer-controlled class 1 testing machine (Instron, Darmstadt, Germany), with a constant speed of 10 ± 1 mm/min. (Figure 3b). The samples were glued into 200 mm × 200 mm metal holders. The tension force values were recorded until damage. Bond strength *σ_T_* was calculated according to (1) and expressed in kPa. In each series, ten samples were tested, and the total was 160 samples.

(1) $$\sigma_{T}=\frac{F_{Tmax}}{A}$$

where: *F_Tmax_*—maximum tensile force, in kN; *A*—bonded area, expressed in m^2^.

### 2.7. Shear Strength and Shear Modulus

For shear strength and shear modulus test, OSB, FGB and CPB, as described in point 2.3, were used. 140 mm × 100 mm samples were cut from them. For each type of substrate, three series of samples were prepared—each under different thermal and moisture conditions (Table 4). The high temperature and low relative humidity (25 ± 2 °C, 30 ± 5%) and high temperature and high relative humidity (25 ± 2 °C, 90 ± 5%) were used, as well as a low temperature (5 ± 2 °C) at the resulting RH.

**Figure 4:**
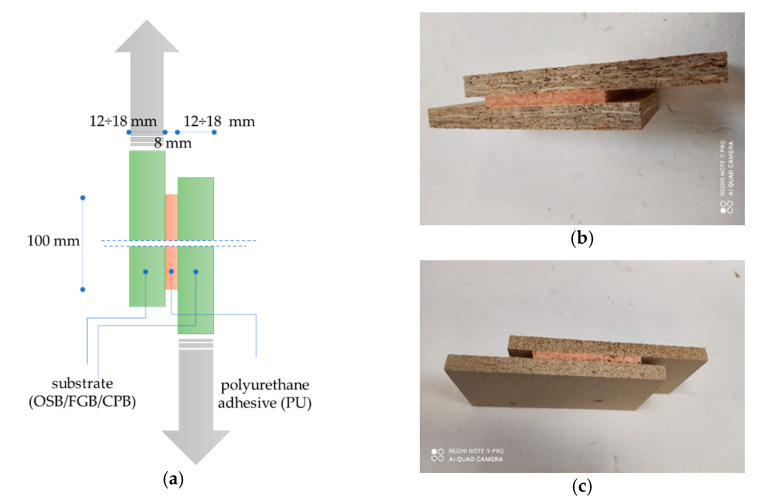
Samples for shear strength and shear modulus test: (**a**) sample scheme, (**b**) OSB/25/30 series sample and (**c**) CPB/25/30 series sample.

OSB, FGB and CPB boards were stored for 24 h under the conditions programmed for the adhesive application. The adhesive itself was stored for 24 h in laboratory conditions. The adhesive was applied to one of the plates, creating a serpentine pattern. At 180 ± 10 s, the boards were joined together to form sets with distance pieces and screw clamps in which the adhesive bond was 8 ± 1 mm thick. The samples were cured for 48 h, and then the excess adhesive in the form of spouts was cut off with a knife, forming adhesive bonds with dimensions of 100 mm × 100 mm × 8 mm (Figure 4).

The shear strength test consisted of determining the maximum stresses with the force acting in the plane of the bond, using the test technique according to EN 12090 [45]. Tests were conducted under laboratory conditions immediately after the samples were cured under specific conditions (Table 4). The test was carried out using a computer-controlled class 1 testing machine (Instron, Darmstadt, Germany), with a constant speed of 3 ± 0.5 mm/min. The shear force value until damage and the force-displacement curve was recorded. In each series, ten samples were tested, and the total was 90 samples. Shear strength *τ* was calculated according to (2), expressed in kPa.

(2) $$\tau =\frac{F_{\tau max}}{A}$$

where: *F_τmax_*—maximum shear force, in kN; *A*—a cross-section of the adhesive bond, in m^2^.

Shear modulus G was calculated according to (3), expressed in kPa.

(3) $$G=\frac{d\cdot \;tan\alpha }{A}$$

where: *d*—adhesive bond thickness, in m; *A*—a cross-section of the adhesive bond, in m^2^; and *tanα-* tangent of the angle of inclination of the straight-line segment of the curve showing the force-displacement relation, in kN/m.

## 3. Results and Discussion

### 3.1. Scanning Electron Microscopy (SEM)

The microstructure is one of the most notable factors that may affect polyurethane adhesive foam properties [40]. The structure, especially cell size and type, depends on the process parameters such as temperature, humidity and viscosity of mixture during foaming [40,46,47]. In general, foam’s physical and mechanical properties depend not only on the rigidity of the polymer matrix but are also related to the cellular structures [36,40]. The closed cells’ size and shape are essential parameters of the foam’s cellular structure, directly affecting the polyurethane foam’s mechanical properties [36].

**Figure 5:**
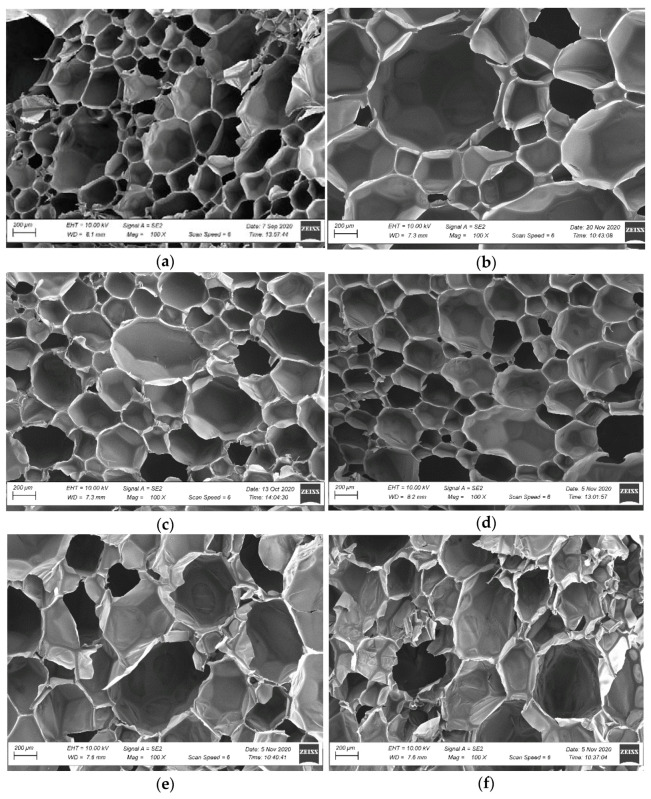
Microstructure of the cross-sectional surface of polyurethane adhesives in the bonds (magnification 100×) (**a**) OSB/23/50/8, (**b**) OSB/23/50/15, (**c**) OSB/25/30/8, (**d**) OSB/5/-/8, (**e**) OSB/25/90/8 and (**f**) OSB/25/90/8.

The microstructure of the polyurethane adhesive foam was analysed by SEM to determine the effect of simulated use conditions on its morphology. The SEM micrographs showed in Figure 5a,c,d revealed a homogeneous microstructure with a closed cell porosity well distributed within the polyurethane matrix. The adhesive structure in 8 mm thick bonds, at the adhesive application in laboratory conditions (Figure 5a), at high temperature and low humidity (Figure 5c) and at low temperature (Figure 5d) is characterised by relatively homogenous pore size. Single pore cells have regular tetraoval shape and comparable size in all three series. Although cells with a diameter lower than 300 μm prevail, individual cells with 500 μm diameter were observed as well. The adhesive cells applied at high temperature and high humidity were noticeably larger (Figure 5e,f). The predominant cells were about 450 μm and larger in diameter. The above can be attributed to the differences in the foaming conditions of polyurethane. An increase in the substrate’s water content intensifies the foaming process, leading to increased cell size [20]. Deformed cell structures were also observed.

The microstructure of the 15 mm thick bond, developed in laboratory conditions (Figure 5b), was also observed. The increase in the bond thickness from 8 mm to 15 mm leads to a loss in the structure homogeneity. The images show a fraction of small pores surrounding individual large cells. Both the cell’s size and shape are important for polyurethane foam’s mechanical properties [40]. A non-homogeneous structure with inclusions of large pores may significantly decrease the mechanical properties.

### 3.2. Apparent Density

**Figure 6:**
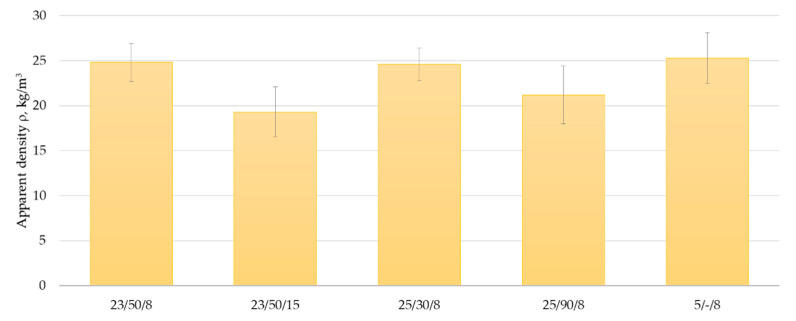
Apparent density of PU adhesive in bonds made under different thermal and moisture conditions. Error bars show standard deviation values.

As it has been already determined in [40], the apparent density of polyurethane foam is among the key parameters, significantly affecting the product’s physical and mechanical properties. The test results summarised in Figure 6 reveal that cured polyurethane adhesive in 8 mm and 15 mm thick bonds were characterised by apparent density from 19.3 kg/m^3^ to 25.3 kg/m^3^. In general, the polyurethane foam’s apparent density depends on the cellular structure [35,37]. Structures with larger cells are characterised by lower apparent density [37,40], which is confirmed by the study results. The highest densities were obtained for the samples taken from 8 mm thick bonds developed in laboratory conditions, at high temperature and low relative humidity, as well at low temperature, amounting to 24.8 kg/m^3^, 24.6 kg/m^3^ and 25.3 kg/m^3^, respectively. According to the description, the adhesive structure in the bonds developed in the abovementioned conditions was homogenous, the cells were uniform and well-defined, and their diameter was up to 300 µm (Figure 5a,c,d). The adhesive density in 15 mm thick bonds and in 8 mm thick bonds formed at high temperature and high relative humidity, for which a non-uniform structure and the presence of cells with ca. 350 µm diameter was observed (Figure 5b,e,f), was lower and amounted to 19.3 kg/m^3^ and 21.2 kg/m^3^, respectively. As expected, the adhesive density in the bonds was higher than the density determined for a free-foamed product and amounted to 18 ± 2 kg/m^3^ (Table 2). The cells in the freely applied products reach higher diameters than under limited product expansion conditions [13].

A literature review revealed that the apparent density of polyurethane foam varies depending on the concentration of water as a blowing agent. The apparent density decreases with the increasing blowing agent content [20,39,40]. It was determined [40] that the polyurethane foam density decreased from 116 kg/m^3^ to 42 kg/m^3^ as the water content increased from 0.1 to 3.0 phr. The same observation was made during studies of closed-cell rigid polyurethane foams based on low functionality polyols [39]. A similar trend was observed in this study. The apparent density of the adhesive in the bonds made at low humidity (25 ± 2 °C, 30 ± 5%) was 15% higher than that for the adhesive in the bonds made at the same temperature but high humidity (25 ± 2 °C, 90 ± 5%).

### 3.3. Mechanical Properties

As mentioned, before their launch, construction products are verified for the building structure’s meeting seven basic requirements, according to CPR [21]. In reference to ETICS, the bond strength, shear strength and shear modulus of the bond adhesive are among the essential requirements that determine the fulfilment of the fourth basic requirement, ‘safety in use’ [5,6,25].

**Figure 7:**
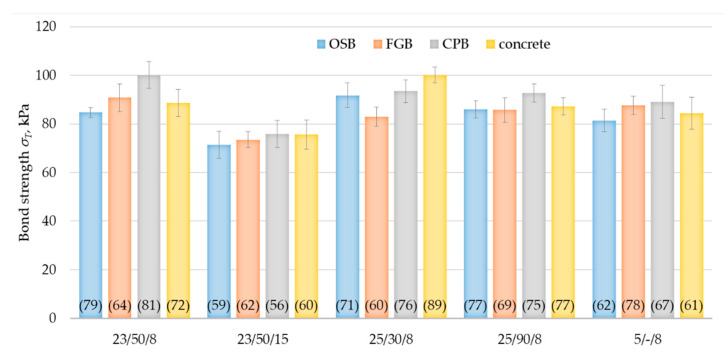
Bond strength results for PU adhesive bonds made under different thermal and moisture conditions. Error bars show standard deviation values. The minimum value for the series is given in brackets.

Analysing the bond strength test results presented in Figure 7, it can be concluded that the bonds of the 8-mm-thick polyurethane adhesive for a system with MW and OSB, and FGB and CPB, had a bond strength similar to that of the reference concrete substrate used as a standard in tests of polyurethane adhesives for EPS-based ETICS. For bonds made under laboratory conditions, bond strength was from 85 to 100 kPa, at high temperature and low relative humidity from 83 to 93 kPa, at high temperature and high relative humidity from 85 to 93 kPa, and at low temperature from 81 to 89 kPa, while for systems with concrete substrate it was 89 kPa, 100 kPa, 87 kPa and 84 kPa, respectively. By analysing the minimum values of the bond strength (Figure 7, values in brackets), one might conclude that for 8 mm thick bonds made in laboratory conditions it ranges from 64 to 81 kPa, at high temperature and low relative humidity from 60 to 76 kPa, at high temperature and high relative humidity from 69 to 77 kPa, and at low temperature from 62 to 78 kPa, while for systems with a reference concrete substrate it is 72 kPa, 89 kPa, 77 kPa and 61 kPa, respectively.

As mentioned, the assessment of the suitability of use of ETICS is carried according to EAD 040083-00-0404 [5] and EAD 040089-00-0404 [6]. Comparison of bond strength values, obtained in our experiment, with the criterion specified at [5,6] for polyurethane adhesives in EPS-based ETICS, which is at least 80 kPa for the average value and at least 60 kPa for the minimum value, allows for a conclusion that the analysed solution is characterised by adhesion at the level higher than the mentioned threshold values. The above could be considered as an important indication for a more favourable assessment of the applicability of polyurethane adhesive as a component of a mineral wool-based ETICS. The obtained results are also in line with essential characteristic of polyurethane adhesives for EPS-based ETICS existing on the market [9,10]. To date, no more information in the literature on the performance of polyurethane adhesives in ETICS has been presented. The researchers’ attention has been directed towards cement-based adhesive systems. The results obtained show that polyurethane adhesives bond strength is significantly lower than bond strength between cement-based adhesive and the concrete [5,31,32]. As it has been already determined in [31], bond strength between cement-based adhesive and the concrete, after 28 days under laboratory conditions, can achieve values above 250 kPa. In other works, bond strength at the level to 1000 kPa was noted [33]. The difference may be explained by differences in structure and material nature of the polymer foams and cement-based products [13]. However, as regards bond strength between cement-based adhesive and the concrete after 28 days under laboratory conditions and 2 days in water, bond strength similar to bond strength of polyurethane adhesives [9,10] can be noted. The test of bond strength between cement-based adhesive and the thermal insulation material is performed separately [5,6]. As it has been already determined in [31,32], it depends strongly on the type of insulation material and the model of damage. For EPS systems, values in the range from 120 kPa to 270 kPa and cohesive rupture in the insulation material were achieved [31,33,34]. However, for MW system values in the range from 30 kPa to 80 kPa, cohesive damage in the insulation material was noted [5,9,10].

The effect of bond thickness was prominent in the tests conducted. For the 15 mm thick bonds, noticeably lower bond strength values were obtained than for the 8 mm thick bonds, as expected. The results were 71 kPa for OSB/23/50/15, 73 kPa for FBG/23/50/15, 76 kPa for CPB/23/50/15 and 76 kPa for the reference substrate C/23/50/15 (Figure 7). Therefore, when compared to the bond strength of bonds made under the same conditions but with a thickness of 8 mm, they were lower by 16%, 19%, 24% and 15%, respectively. These differences are due to differences in the adhesive cellular structure [35]. According to the experience of other researchers, in wider bonds carbon dioxide has the ability to form larger bubbles, resulting in a more porous structure [40]. The performed SEM analysis indicates cells less than 300 μm in diameter predominated in the 8 mm bond (Figure 5a). Cells of the adhesive in the 15 mm bond were noticeably larger. The predominant cells were about 450 μm and larger in diameter (Figure 5b) as a previous study showed more porous polyurethane foam may have a lower tensile strength [37]. By comparing the test results for 15 mm thick bonds with the criterion specified for PU adhesives in EPS-based ETICS of at least 80 kPa [5,6], it can be seen that significantly lower values were obtained. In this case, consideration should be given to limiting the use of the adhesive on substrates where no irregularities are necessitating the use of 15 mm thick bonds. Taking into account that the deviation from the flatness of OSB, FBG and CPR is usually less than 5 mm [41,42], this condition does not pose a serious problem.

A correlation between the bond strength and the apparent adhesive density was noted. As has been already determined [40], higher apparent density of polyurethane foam resulted in higher mechanical properties. A similar effect was observed in this study. The highest bond strength was obtained for bonds developed in laboratory conditions at high temperature and low relative humidity, as well as at low temperature, whose densities were 24.8 kg/m^3^, 24.6 kg/m^3^ and 25.3 kg/m^3^, respectively. No such regularity was observed for bonds developed at high temperature and high relative humidity.

**Figure 8:**
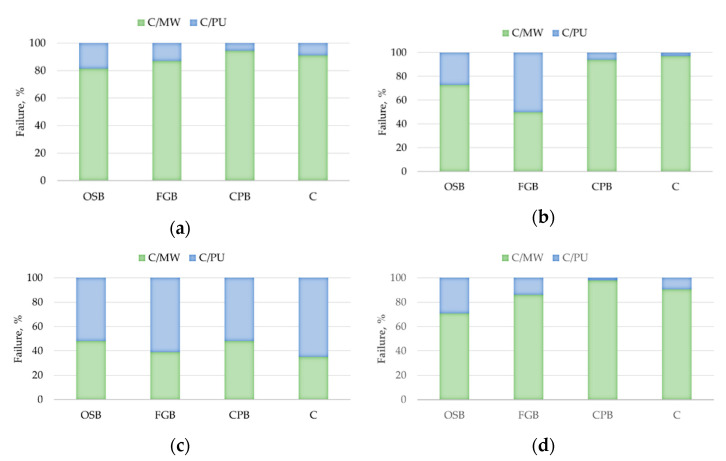
Model of damage—average values for the series: (**a**) 23/50/8, (**b**) 25/30/8, (**c**) 25/90/8 and (**d**) 5/-/8 (C/MW—cohesive damage within the MW, C/PU—cohesive damage within the PU adhesive).

**Figure 9:**
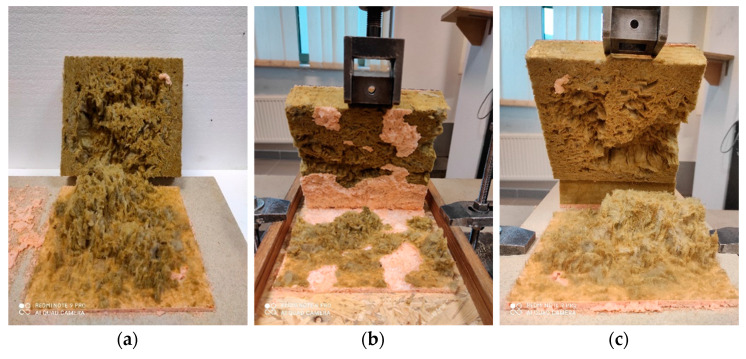
Illustration of the model of damage of 8 mm thick bonds (**a**) CPB/23/50/8 series sample—C/MW damage, (**b**) OSB/23/50/8 series sample—C/MW damage combined with C/PU damage and (**c**) CPB/23/50/8 series sample—C/MW damage (C/MW—cohesive within the MW, C/PU—cohesive within the PU adhesive).

**Figure 10:**
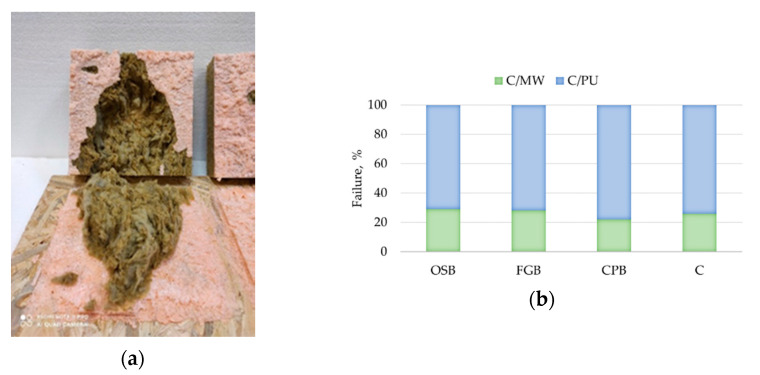
Illustration of the damage of 15 mm thick bonds (**a**) OSB/23/50/15 series sample, (**b**) average values for individual series (C/MW—cohesive damage of the MW, C/PU—cohesive damage of the PU adhesive).

Analysis of the cross-sections of the samples after testing clearly indicates the cohesive model of the damage. For the 8 mm thick adhesive bonds made under laboratory conditions, high temperature and low relative humidity, as well as low temperature, damage within the MW was predominant. In these series, the average proportion of damage within the MW was up to 80 to 95% (Figure 8a and Figure 9a–c), 50 to 95% (Figure 8b) and 70 to 90% (Figure 8d), respectively. The above indicates that the bond strength exceeded the perpendicular tensile strength of the thermal insulation material itself. A similar effect was observed for MW-based ETICS with cement-based adhesive [32]. Cohesive damage was observed also for bonds made at high temperature and high relative humidity but with predominant damage within the polyurethane adhesive. The proportion of damage in MW ranged from 35 to 48% (Figure 8c). Cohesive damage within the polyurethane adhesive was also recorded for 15 mm thick adhesive bonds (Figure 10a). The proportion of MW damage ranged from 22% to 28%, which is noticeably lower than for the 8 mm thick bonds where it ranged from 80 to 95% (Figure 10b). Again, these differences can be explained by the differences in the cellular structure of adhesive. More porous polyurethane adhesive may obtain lower tensile strength [35,37,39].

There was no significant effect of sheathing type (OSB, GFB and CPB) on bond strength. The same observation was made during studies of cement-based adhesive [34]. In the series prepared under laboratory conditions, the highest value was for CPB/23/50/8—100 kPa and the lowest was for OSB/23/50/8—85 kPa; in the series prepared at high temperature and low relative humidity, the highest value was for CPB/25/30/8—93 kPa and the lowest for FGB/25/30/8—83 kPa; for series prepared at high temperature and high relative humidity, the highest value was for CPB/25/90/8—93 kPa and the lowest for FGB/25/90/8—85 kPa; and for series prepared at low temperature, the highest value was for CPB/5/-/8—89 kPa and the lowest for OSB/5/-/8—81 kPa. The above indicates that the performance evaluation process may consider limiting the number of test runs to one type of sheathing.

No effect of substrate type on the model of the damage was observed. The GFB/25/30/8 series highlighted samples slightly in this respect, for which, as for the OSB/23/50/8 series, the proportion of damage within the polyurethane adhesive was recorded at 50%, while for samples on other substrates, it ranged from 5 to 25%. No such regularity was observed in the other test series.

Summarising the experimental data on bond strength obtained in this study, it can be stated that the tested polyurethane adhesive showed satisfactory adhesion to both mineral wool (MW) and boards typical for sheathing of walls of frame structure—oriented strand boards (OSB), fibre-reinforced gypsum boards (FGB) or cement-bonded particleboards (CPB). The cohesive property of the damage, predominantly within the thermal insulation material, indicates that the polyurethane adhesive bonds’ bond strength may exceed the perpendicular tensile strength of the thermal insulation material itself. It should also be noted that mineral wool lamella, without coatings or facing, with a perpendicular tensile strength ≥80 kPa (TR80) was used in the tests. The factor determining the bond strength was, as expected, the thickness of the adhesive bond. Increasing the thickness from 8 mm to 15 mm resulted in a decrease of approximately 20% in bond strength. The effect of the thermal and moisture conditions under which the bonds were made and cured was also outlined. The lowest values of bond strength were recorded for the series prepared at low temperature, next at high temperature and high relative humidity, high temperature and low relative humidity, and the highest at laboratory conditions. In contrast, it should be noted that only in the series prepared at high temperature and high relative humidity the damage of the polyurethane adhesive predominated. In contrast, the damage of MW predominated in the other cases, so the decisive influence on the values obtained was the properties of the thermal insulation material. No effect of substrate type (OSB, FGB, CPB or concrete) on bond strength was observed.

**Figure 11:**
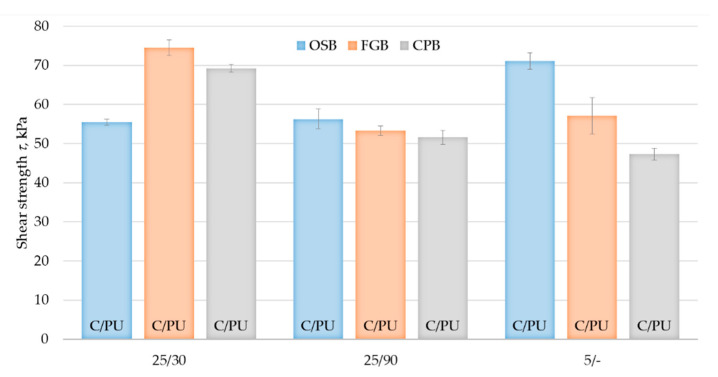
Shear strength tests results of bonds made under different thermal and moisture conditions. Error bars show standard deviation values. Data supplemented with a description of the damage: C/PU—damage of cohesion in PU adhesive.

**Figure 12:**
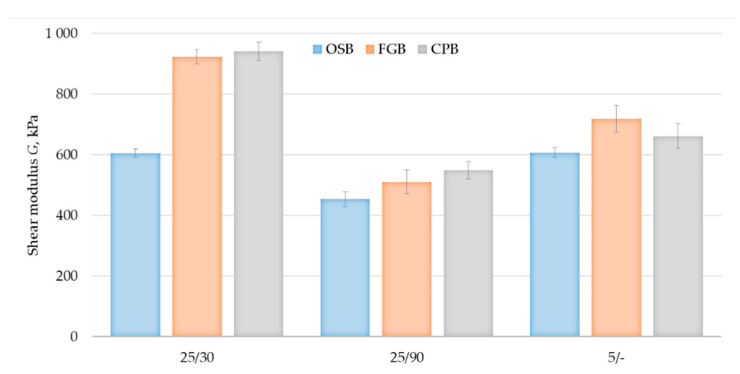
Shear modulus tests results of bonds made under different thermal and moisture conditions (error bars show standard deviation values).

Shear strength and shear modulus were analysed in terms of the influence of the type of substrate, taking into account the boards standard for the sheathing of timber frame walls and the adhesive thermal and humidity conditions bonds. The shear strength values are shown in Figure 11, and the shear modulus values are shown in Figure 12. The highest values of the considered properties were recorded for the samples prepared at high temperature and low relative humidity, obtaining shear strength of 55 kPa for OSB/25/30 series, 75 kPa for FGB/25/30 and 69 kPa for CPB/25/30 and shear modulus of 605 kPa, 920 kPa and 940 kPa, respectively. The bonds made at high temperature with high relative humidity showed significantly lower values concerning their properties, which may be dictated by the difference in the adhesive cell structure (Figure 5). Shear strength of 56 kPa for OSB/25/90 series, 52 kPa for FGB/25/90 series and 52 kPa for CPB/25/90 series was obtained, while for shear modulus, it was 455 kPa, 510 kPa and 590 kPa, respectively. The properties of bonds made at low temperature were of average values, except for the shear strength of the OSB/5/- bonds where a value of 71 kPa was recorded, while it was 57 kPa for FGB/5/and 47 kPa for CPB/5/-. Shear modulus was 610 kPa, 720 kPa and 660 kPa, respectively. All tested samples proved to be vulnerable to cohesive damage, in 100% within the adhesive bond, which confirms the high adhesion of polyurethane adhesive to all considered substrates—OSB, FGB and CPB—recorded bond strength tests. No significant effect of substrate type on the properties considered was observed.

The shear strength values obtained in this study were slightly lower than those approved for typical adhesives intended for use in EPS-based ETICS [9,10]. The shear modulus values were close to those indicated in [9] and significantly higher than those specified in [10]. It should also be noted that within the framework of the above-mentioned ETA procedures, the tests of bonds made under laboratory conditions on standard particleboards were carried out. Shear strength and shear modulus, according to both EAD 040083-00-0404 [5] and EAD 040089-00-0404 [6] guidelines, should be considered as a property of adhesive bond that can be used in the insulation design process.

## 4. Conclusions

The analysis of the experimental data obtained in this study proves that there are indications for an upbeat assessment of the applicability of polyurethane adhesive as a component of a mineral wool-based ETICS, intended for fixing thermal insulation material to the sheathing of walls with the timber frame structure.

It has been shown that polyurethane adhesive can achieve satisfactory adhesion to mineral wool lamella (TR80) without coatings and facing in the form of fabric, veil, film, etc. Bond strength of bonds made in thermal and moisture conditions limited for the considered application, with a bond thickness of 8 mm, achieved a satisfactory for ETICS value above 80 kPa.

It was also found that polyurethane adhesive has good adhesion to boards typical for timber frame wall sheathing—oriented strand boards (OSB), fibre-reinforced gypsum boards (FGB) or cement-bonded particleboards (CPB). No significant effect of board type on bond strength, shear strength and shear modulus was determined.

The methodology of testing the performance of polyurethane adhesives intended for fixing mineral wool boards requires the analysis of the specifics of the polyurethane applied on site and the thermal insulation material and the sheathing boards. The test shows that the introduction of appropriate modifications to standard procedures established for EPS-based ETICS makes it possible to obtain data indispensable for assessing the performance of adhesives intended for MW-based ETICS.

Taking into account the diversity of both polyurethane adhesives and mineral wool face finishes, the authors intend to continue work focused on the aspect of adhesion. Furthermore, verification of the performance of MW-based ETICS made with the use of polyurethane adhesive is planned on facade models, including all components of the system.

## Figures and Tables

**Table 1:** Properties of mineral wool (MW) boards used in ETICS.

Symbol Present in Item Code	Property	Type of Boards	
		Lamella	Standard
-	Reaction-to-fire performance	Class A1 or A2	
-	Thermal resistance	value declared by the manufacturer according to EN 13162	
T5	Thickness tolerance	−1% or −1 mm^1^ +3 mm	at least −3% or −3 mm^1^ +5% or +5 mm^2^
DS(70, -)	Dimensional stability (48 h, T70 °C)		
	thickness change Δε_d_	≤1%	
	length and width change Δε_b_	≤1%	
DS(70,90)	Dimensional stability (48 h, T70 °C)		
	thickness change Δε_d_	≤1%	
	length and width change Δε_b_	≤1%	
WS	Water absorption after 24 h of partial immersion	≤1 kg/m^2^	
WL(P)	Water absorption after 28 days of partial immersion	≤3 kg/m^2^	
MU1	Water vapour diffusion resistance factor	1	
TR	Perpendicular tensile strength	≥80 kPa	≥7.5 kPa

**Table 2:** Polyurethane adhesive working properties.

Property	Value
Minimum ^1^ limit temp. of application ^2^	5 °C
Maximum ^1^ limit temp. of application ^2^	25 °C
Recommended temp. of storage	>20 °C
Density (at free foaming, after curing)	18 ± 2 kg/m^3^
Post-expansion	2 mm
Curing time	6 min
Cutting time	20 min

^1^ Declared by the manufacturer, ^2^ temperature of the substrate, insulation material and environment.

**Table 3:** Conditions of sample preparation for bond strength testing.

Designation of Test Series	Type of Substrate	Seasoning Conditions				Conditions of Adhesive Application		Conditions of Curing	
		Substrate		Adhesive					
		T, °C	RH, %	T, °C	RH, %	T, °C	RH, %	T, °C	RH, %
8 mm thick bond									
OSB/23/50/8	OSB	23 ± 2	50 ± 5	23 ± 2	50 ± 5	23 ± 2	50 ± 5	23 ± 2	50 ± 5
OSB/25/30/8		25 ± 2	30 ± 5			25 ± 2	30 ± 5	25 ± 2	30 ± 5
OSB/25/90/8		25 ± 2	90 ± 5			25 ± 2	90 ± 5	25 ± 2	90 ± 5
OSB/5/-/8		5 ± 2	- ^1^			5 ± 2	-	5 ± 2	-
FGB/23/50/8	FGB	23 ± 2	50 ± 5	23 ± 2	50 ± 5	23 ± 2	50 ± 5	23 ± 2	50 ± 5
FGB/25/30/8		25 ± 2	30 ± 5			25 ± 2	30 ± 5	25 ± 2	30 ± 5
FGB/25/90/8		25 ± 2	90 ± 5			25 ± 2	90 ± 5	25 ± 2	90 ± 5
FGB/5/-/8		5 ± 2	-			5 ± 2	-	5 ± 2	-
CPB/23/50/8	CPB	23 ± 2	50 ± 5	23 ± 2	50 ± 5	23 ± 2	50 ± 5	23 ± 2	50 ± 5
CPB/25/30/8		25 ± 2	30 ± 5			25 ± 2	30 ± 5	25 ± 2	30 ± 5
CPB/25/90/8		25 ± 2	90 ± 5			25 ± 2	90 ± 5	25 ± 2	90 ± 5
CPB/5/-/8		5 ± 2	-			5 ± 2	-	5 ± 2	-
C/23/50/8	Concrete	23 ± 2	50 ± 5	23 ± 2	50 ± 5	23 ± 2	50 ± 5	23 ± 2	50 ± 5
C/25/30/8		25 ± 2	30 ± 5			25 ± 2	30 ± 5	25 ± 2	30 ± 5
C/25/90/8		25 ± 2	90 ± 5			25 ± 2	90 ± 5	25 ± 2	90 ± 5
C/5/-/8		5 ± 2	-			5 ± 2	-	5 ± 2	-
15 mm thick bond									
OSB/23/50/15	OSB	23 ± 2	50 ± 5	23 ± 2	50 ± 5	23 ± 2	50 ± 5	23 ± 2	50 ± 5
FCB/23/50/15	FCB								
CPB/23/50/15	CPB								
C/23/50/15	Concrete								

^1^ resulting (ca. 30 ± 5%).

**Table 4:** Conditions for preparation of samples for shear strength and shear modulus test.

Designation of Test Series	Type of Substrate	Seasoning Conditions				Conditions of Adhesive Application		Conditions of Curing	
		Substrate		Adhesive					
		T, °C	RH, %	T, °C	RH, %	T, °C	RH, %	T, °C	RH, %
OSB/25/30	OSB	25 ± 2	30 ± 5	23 ± 2	50 ± 5	25 ± 2	30 ± 5	25 ± 2	30 ± 5
OSB/25/90		25 ± 2	90 ± 5			25 ± 2	90 ± 5	25 ± 2	90 ± 5
OSB/5/-		5 ± 2	- ^1^			5 ± 2	-	5 ± 2	-
FGB/25/30	FGB	25 ± 2	30 ± 5	23 ± 2	50 ± 5	25 ± 2	30 ± 5	25 ± 2	30 ± 5
FGB/25/90		25 ± 2	90 ± 5			25 ± 2	90 ± 5	25 ± 2	90 ± 5
FGB/5/-		5 ± 2	-			5 ± 2	-	5 ± 2	-
CPB/25/30	CPB	25 ± 2	30 ± 5	23 ± 2	50 ± 5	25 ± 2	30 ± 5	25 ± 2	30 ± 5
CPB/25/90		25 ± 2	90 ± 5			25 ± 2	90 ± 5	25 ± 2	90 ± 5
CPB/5/-		5 ± 2	-			5 ± 2	-	5 ± 2	-

^1^ resulting (ca. 30 ± 5%).

## Data Availability

The data presented in this study are available on request from the corresponding author.
